# Population health management in Belgium: a call-to-action and case study

**DOI:** 10.1186/s12913-023-09626-x

**Published:** 2023-06-20

**Authors:** Betty Steenkamer, Bert Vaes, Ernst Rietzschel, John Crombez, Sabina De Geest, Fabian Demeure, Marijke Gielen, Michel P. Hermans, Stefan Teughels, Peter Vanacker, Thierry van der Schueren, Steven Simoens

**Affiliations:** 1 Stichting Gezondheidscentra Eindhoven - STROOMZ NL, Eindhoven, the Netherlands; 2 Department of Public Health and Primary Care, KU Leuven, Leuven, Belgium; 3 Department of Internal Medicine & Paediatrics, Ghent University, Ghent, Belgium; 4 Biobanking and Cardiovascular Prevention, Ghent University Hospital, Ghent, Belgium; 5 Architecture of a Qualitative, Sustainable and Inclusive Health system (AQSIH), Ghent University Hospital, Ghent, Belgium; 6 Department of Public Health, University of Basel, Basel, Switzerland; 7 Cardiology Department, CHU UCL Mont-Godinne, Namur, Belgium; 8 Independent Health Insurance Funds, Brussels, Belgium; 9 Endocrinology & Nutrition, Cliniques universitaires St-Luc, Brussels, Belgium; 10 Medical School, Catholic University of Louvain, Brussels, Belgium; 11 Domus Medica, Antwerp, Belgium; 12 Department of Neurology, AZ Groeninge, Kortrijk, Belgium; 13 Department of Neurology, University Hospitals Antwerp, Antwerp, Belgium; 14 Department of Translational Neuroscience, University of Antwerp, Antwerp, Belgium; 15 Société Scientifique de Médecine Générale, Brussels, Belgium; 16 Department of Pharmaceutical and Pharmacological Sciences, KU Leuven, Leuven, Belgium

**Keywords:** Population health management, Barriers, Lessons, Implementation, Belgium, Atherosclerotic cardiovascular disease

## Abstract

**Background:**

Although there are already success stories, population health management in Belgium is still in its infancy. A health system transformation approach such as population health management may be suited to address the public health issue of atherosclerotic cardiovascular disease, as this is one of the main causes of mortality in Belgium. This article aims to raise awareness about population health management in Belgium by: (a) eliciting barriers and recommendations for its implementation as perceived by local stakeholders; (b) developing a population health management approach to secondary prevention of atherosclerotic cardiovascular disease; and (c) providing a roadmap to introduce population health management in Belgium.

**Methods:**

Two virtual focus group discussions were organized with 11 high-level decision makers in medicine, policy and science between October and December 2021. A semi-structured guide based on a literature review was used to anchor discussions. These qualitative data were studied by means of an inductive thematic analysis.

**Results:**

Seven inter-related barriers and recommendations towards the development of population health management in Belgium were identified. These related to responsibilities of different layers of government, shared responsibility for the health of the population, a learning health system, payment models, data and knowledge infrastructure, collaborative relationships and community involvement. The introduction of a population health management approach to secondary prevention of atherosclerotic cardiovascular disease may act as a proof-of-concept with a view to roll out population health management in Belgium.

**Conclusions:**

There is a need to instill a sense of urgency among all stakeholders to develop a joint population-oriented vision in Belgium. This call-to-action requires the support and active involvement of all Belgian stakeholders, both at the national and regional level.

## Background

Demographic and social changes such as an aging population, increasing multi-morbidity and technological developments, and the changing needs that society places on care and support, require far-reaching adjustments to the way in which care and support are offered [1–4]. At the same time, financial stringencies are increasing and available workforces are limited. As a result, transformative approaches such as Population Health Management (PHM) are becoming widespread in health policy and in practice [5, 6].

PHM is a proactive approach in which initiatives use data and risk stratification as the starting point to investigate variation in health outcomes [5, 7, 8]. This approach allows for sharing of insights across organisations and sectors, and for a better understanding of what is happening in communities, i.e. where needs are unmet, or where people are most at risk of negative health outcomes now and in the future [4–9]. These insights enable PHM initiatives to collaboratively look for solutions, and subsequently implement, evaluate and refine corresponding interventions. PHM initiatives play a significant role in the movement to improve population health and quality of care, while at the same time reducing cost growth as well as promoting well-being and reducing health inequalities across an entire population (nowadays called the quintuple aims) [4, 5, 10–12]. They recognize that to implement large-scale transformations, a wide range of organisations have to work together and explore which strategies will not only strengthen connections and integrate services across public health, health care, social care and community services, but also transform how health care is delivered in order to address the full range of health determinants and build more healthy communities [5, 10–13].

PHM has been more and more embraced in policy and practice in many countries such as the Dutch ‘PHM pioneer sites’ and the implementation of the national policy program ‘The right care at the right place’ in the Netherlands [5, 13]. In England, the National Health Service Long Term Plan [14] which has led to PHM Integrated Care Systems, has recently been updated in the call-to-action for a PHM approach as a response against COVID-19 [15]. Another example is the implementation of a PHM approach towards secondary prevention in patients with atherosclerotic cardiovascular disease (ASCVD), involving the collaboration between the UK Government, the National Health Service, the Academic Health Science Network and Novartis UK [16]. Although a PHM approach targeting ASCVD patients has not (yet) been implemented in Belgium, malignant tumors and ASCVD are the main causes of mortality in Belgium, accounting together for more than half of all deaths in both sexes [17]. The conglomerate of ASCVD risk factors (which is also a common soil for chronic kidney disease, tumours, lung disease and other chronic diseases), and resulting ASCVD remain a public health issue.

The literature proposes a number of principles, building blocks, and key activities that underpin PHM. It is advocated that the development of a PHM approach is guided by principles that create commitment between stakeholders, attain understanding of respective values and roles, set conditions for accountability, gain political support for PHM, introduce financial incentives that sustain the quintuple aims, promote a permanent improvement cycle supported by a data and knowledge infrastructure, ensure community engagement and stakeholder representation [5, 18]. When setting up an interdisciplinary collaboration around PHM, infrastructural building blocks need to be put in place that relate to social forces, resources and technologies, finance, relations, regulations, market, leadership, accountability, and community engagement [5, 11]. Finally, a PHM approach requires the following key activities: (a) population identification; (b) triple aim assessment; (c) risk stratification and intervention selection; (d) citizen-centered interventions; (e) quintuple aim impact evaluation; and (f) a quality improvement process [5, 6, 8, 19].

Despite policy measures of the Belgian government to move from a supply-based to a needs-based approach [20, 21], the implementation of PHM in Belgium is still in its infancy [22]. Therefore, the aim of this study is to raise awareness about PHM in Belgium by eliciting barriers and recommendations for its implementation as perceived by local stakeholders. These barriers and recommendations are then exemplified in the context of a case study focusing on secondary prevention in patients with ASCVD. Finally, a roadmap is provided with next steps to roll out PHM in Belgium. Although this study focuses on Belgium, barriers and recommendations may be applicable to the implementation of PHM in other countries.

## Methods

### Study design

Two virtual focus group discussions were held between October and December 2021 to gather insights into barriers and recommendations for the implementation of PHM in Belgium. The methodology of focus group discussion allows for interaction between participants given that the opinion of a participant can be put forward and be challenged by others [23]. The moderator (SS) drew on this interaction to find a common ground between diverse stakeholders.

### Sampling

Eleven high-level decision makers in medicine, policy and science were purposefully selected to participate in both focus group discussions. As indicated in Table 1, most participants had multiple professional backgrounds and were primary or secondary care physicians, worked for physician professional associations or a health insurance fund, and were academics with an interest in PHM or health system architecture. The purposive sampling method [24] was chosen with a view to enroll representatives of the diverse stakeholders likely to be involved in the initiation of PHM in Belgium or individuals who have PHM expertise. Both participants from the Flemish-speaking and French-speaking parts of Belgium were enrolled.

**Table 1:** Professional background of focus group participants

	Physician	Academic	Professional society or policy experience	Health insurance fund
1				X
2	X		X	
3	X		X	
4	X	X	X	
5		X		
6		X	X	
7	X	X	X	
8	X	X	X	
9	X	X	X	
10	X	X	X	
11	X	X		

### Focus group guide

A semi-structured guide was used to anchor the discussions and to ensure that all different aspects which can influence PHM development, were included [5, 6, 8, 11, 18, 19]. This guide was based on a literature review examining the current state of the art with respect to PHM, and was specifically informed by guiding principles, building blocks, and key activities that give insight into the underlying barriers and recommendations for the development, implementation and evaluation of a PHM approach (see Introduction).

### Focus group sessions

The focus group sessions were designed to take place sequentially with the second session building on the first session as explained below. In the first focus group session, the literature review was presented to participants by a PHM expert (BS). This was followed by a discussion of barriers and recommendations of PHM development in Belgium, based on insights written down by each participant in a MIRO board (i.e. an online collaborative whiteboard). Overall insights which emerged from this first focus group session, were validated by all participants during a second session following some final changes and additions.

Secondary prevention of ASCVD was chosen as a case for PHM development in the second focus group session. This is because ASCVD is a public health issue and a disruptive access model such as PHM which aims to prevent, treat, and support adherence in ASCVD patients, could reduce heart attacks and strokes. Also, ASCVD patients are a well-defined, substantial and relatively homogeneous population, which makes it easier to identify in databases and to reach consensus on PHM development among health care providers. Although we acknowledge that PHM is geared at the general population, this case study in ASCVD patients may serve as a proof-of-concept and the resulting experience could subsequently be applied to a broader population.

With a view to prepare a discussion on the case study, three high-level decision makers (BS, BV, ER) with expertise in PHM, primary and secondary ASCVD care, respectively, identified barriers and recommendations regarding the six key activities [8] that make up the PHM program focusing on secondary prevention in ASCVD patients. In the second focus group session, two break-out groups discussed the practical development of a PHM program focusing on secondary prevention in patients with ASCVD, after which these results were discussed among all focus group participants.

### Data analysis

With permission of all participants, both focus group sessions were recorded and responses were transcribed *ad verbatim*. An inductive thematic analysis (by BS) was carried out of these qualitative data, involving the subsequent steps of data familiarization, development and review of themes, mapping and interpretation by theme, and selection of appropriate quotes [25]. All focus group participants validated the results of the data analysis.

## Results

### General barriers and recommendations for PHM implementation in Belgium

Participants identified seven inter-related barriers and recommendations towards the implementation of PHM in Belgium (see Table 2).

**Table 2:** Barriers and recommendations for implementing population health management in Belgium

*Barriers*	*Recommendations*
Complexity of the Belgian political system leads to lack of urgency for PHM development	Fragmentation of responsibilities needs to be homogenized in addition to encouraging a sense of urgency, with a view to work towards a joint population-oriented vision and a sustainable health and wellbeing system
Lack of financial incentives	New financial arrangements and payment models are necessary that instigate PHM development
Lack of awareness and shared responsibility/accountability for the health of the population	Start from a joint population-oriented vision at all levels Gather insight into population needs with a view to create awareness and shared responsibilities in delivering prevention, care and support for the people in the community
Lack of a learning environment to stimulate PHM development	Develop a learning environment that supports system improvements based on data Collaboratively gather and share data, support a performant data and knowledge infrastructure at the organizational and the regional-national level
Lack of political support and hindering laws and regulations	Engage with national/regional governments Involve knowledge institutions for policy advice
Insufficient communication and trust between providers and stakeholder organizations to work towards PHM	Develop collaborative relationships at and between all levels Negative consequences for existing norms, values, roles and accountability need to be addressed on the national and regional stakeholder level
Lack of patient/community involvement	Focus on patient and population needs and community empowerment is necessary for better awareness of ways to improve health and wellbeing

Note: PHM = population health management.

#### Complexity of the Belgian political system leads to lack of urgency for PHM development

Participants stated that the three policy levels within the Belgian political system, i.e. federal authorities, federated entities and local authorities, lead to fragmented responsibilities. These affect the preparedness for change as different aspects of the continuum of services (public health, healthcare, social care and community services) are governed by policies formulated at different levels of government [26]. According to participants, this fragmented policy has hindered the development of a shared population-oriented long-term vision and a learning health system working towards sustainability and wellbeing. For instance, policies regarding health promotion and disease prevention are the responsibility of the federated entities, while healthcare is a federal responsibility. Participants indicated that fragmented responsibilities for health promotion/disease prevention and curative medicine influences the allocation of resources. Investments in health promotion/disease prevention activities by federated entities are expected to lead to cost savings at the level of federal authorities, so if federated entities do not receive any financial benefits, they may become disengaged to invest in such activities.

'*The profits to be gained through prevention are foremost by federal authorities…we need a homogenized responsibility for prevention*.'

'*Preventive medicine needs to be more integrated within the health care system. Belgium still has low success rates of smoking cessation or weight loss. Healthy lifestyle options rely too much on personal decisions instead of proactive policies… resources are mainly focused on treatment and cure of sick people instead of prevention of illness and keeping people healthy.'*

Furthermore, participants mentioned that due to the overabundance of governance structures and levels, negotiation and policy making processes take a long time. Moreover, according to participants, history has shown that these different levels have led to competitive behaviors that are difficult to reconcile, as the different entities often set their own priorities to achieve the best outcomes for their policy in order to appear successful. Participants stated that these self-serving political considerations and fragmented responsibilities influence the amount of (political) support for large-scale transformative approaches towards healthy communities and a learning health system. Despite recent policies that stimulated integrated care pilot projects [21], scaling up these projects towards a population-based approach is necessary in light of current and future socio-demographic, epidemiological, financial and technological developments in the Belgian health system. Participants stated that a sense of urgency seems to be lacking despite the recent COVID-19 pandemic and that PHM needs to be put on the political agenda with a view to work towards a proactive population-oriented vision and implementation.

’*These fragmented policy levels have hindered a shared long-term population-oriented vision. We need to go to a learning health system that works towards sustainability and wellbeing.’*

'*It doesn’t help that we have nine healthcare ministers for what…eleven and a half million Belgians?'*

Finally, participants emphasized that the steps in policy and practice that have been made, such as the reform of primary health care in Flanders and the pilot projects on integrated care for the chronically ill [21], may facilitate the development of a population needs-based approach.

#### Lack of financial incentives

Provider payment in the Belgian health care system is currently mainly based on fee for service (which incentivizes individual services) and is acute-care driven.

'*From a financial perspective, the system is still reactive, acute-care driven, instead of proactive, prevention driven.'*

Essential elements of the PHM approach such as prevention and targeted action towards quality improvement and reduction of inequalities need to be supported by new financial arrangements. In addition, current financial incentives do not promote cooperation and referral as these potentially negatively influence providers’ income, especially for self-employed health care workers. Some participants worried about the widening of gaps in accessibility of care given that solely target-based financial incentives might trigger physicians not to take care of complex patients.

'*Target-based financial incentives are a double-edged sword.'*

A mix of a fee-for-service model and a capitation-based payment method was therefore seen as important to make steps towards integrating services. Furthermore, cross-sector collaboration (i.e. public health, health and social care and community services) and referral were seen as necessary by participants to ensure a continuum of services and patient/citizen/population-oriented management and delivery of health services instead of the current episodic and provider-oriented system.

#### Lack of awareness and shared responsibility/accountability for the health of the population

Besides a new incentive design, participants stated that data input and knowledge of data technology, data analysis and synthesizing data into meaningful information was necessary to support multidisciplinary responsibility for improvements of population health. These aspects were considered as important for creating awareness of the needs, activities and responsibilities in delivering prevention, care and support.

'*We lack a culture of quality control, being compared to peers in Belgium…we need a good selection of parameters and trust building.'*

Developing a culture for common responsibility in which risks and successes are shared is still difficult due to for instance a fear of benchmarking. Shared responsibility is for example hindered by differences in cut-off values and scores, e.g. academic hospitals only want to be benchmarked with other academic hospitals as their patient population is very different from that of community hospitals.

#### Lack of a learning environment to stimulate PHM development

Participants stated that PHM is a language that policy developers and providers are not familiar with. Policy developers and providers within the health care system need the opportunity to become more acquainted with PHM in order to move beyond a supply-based approach to single diseases towards a population-based approach. How to use available data and insights to segment the population and identify those most at risk of negative health outcomes now or in the future, and to work together with stakeholders and the local population to look for solutions and implement and evaluate these, is not common language in Belgium. Specifically, participants stressed the need for: (a) insight into regional needs, trends and priorities; (b) supporting structures and processes for training of professionals in data management; (c) data flows across organizations and sectors; and (d) measurements and monitoring supported by information flows at management and organizational level. These elements are required in order to identify those who are at risk and to proactively manage prevention, while improving the health of the population. Currently an adequate health information system that allows for sharing of data between different (types of) stakeholder groups (medical and social sector) and patients/care givers in the home environment is lacking. In addition, health care professionals generally have no or little knowledge of data management. Also, participants pointed to the quality of available data which might leave a lot to be desired, as a consequence of a suboptimal registration culture and discipline among providers. According to participants, these issues need involvement of education and knowledge institutions and the government.

#### Lack of political support and hindering laws and regulations

Despite a digital health strategy that has been formulated and implemented by federal authorities and federated entities, participants stated that privacy issues and legislation surrounding data sharing remain unclear. In addition, participants mentioned the under-utilization of existing data sources or lack of access to often fractionated data sources (e.g. between the National Institute for Health and Disability Insurance, health insurance funds, and software platforms).

'*There is a lot of data out there, but we don’t use it. And lack of access to data and fractionated data makes it difficult to gather epidemiological data. The government should be more supportive.'*

'*Quality of data is key, e.g. social/ethnic differences between populations, rich data can show what should be done to prevent multi-morbidity for subpopulations and reduce health inequalities across an entire population.'*

A participant mentioned that rich data can improve precision of decision-making, but questioned if it could lead to prioritizing health needs if these were not backed up by political support at all levels. Furthermore, in light of the discussion whether to pursue a centrally governed electronic health system based on a federated data network or to stimulate free-market competition, some participants were skeptical towards private health management companies being in charge of developing such a system. They also thought that the market potential in Belgium would be too small in order for these private companies to develop and sell their packages.

Furthermore, participants mentioned hindering laws and regulations with regard to task shifting. In light of changing roles of professionals such as specialist nurses within the health care system, participants stated that task shifting is important in order to adapt to evolving patient and population needs. Currently, numerous tasks are performed by overqualified health care providers in Belgium as some procedures are only remunerated when performed by a physician. According to participants, the efficient division of tasks is hampered by laws and regulations on the execution of tasks by health care providers and by reimbursement rules.

#### Insufficient communication and trust between providers and stakeholder organizations to collaboratively work towards PHM

Participants mentioned insufficient communication between providers in the general interest of the patient, specifically within primary care and between primary and hospital care on the one hand, and within rehabilitation and nursing home care on the other hand. According to participants, insufficient communication can lead to lack of accountability after a patient is discharged. For instance, better communication between different health care professionals is necessary in light of (re-)examination of (existing) pharmacotherapies during patient follow-up. In addition, participants stated that better communication between stakeholder organizations could benefit the collaborative process necessary to align measures and actions across an individual’s life course in order to develop healthier communities.

'*Adopt a life-course approach, in which measures are aligned around the life course of an individual, from birth to disease state, e.g. for school children: focus on health literacy and a healthy environment (primordial prevention), later in life: focus on primary prevention, and once a person gets sick: move from population health to an individualized clinical approach. As such, by translating the public health and clinical languages and focusing it on the patient, different stakeholders - public health, general practitioners, specialists - can be brought together.'*

#### Lack of patient/community involvement

Participants stated that patient and community engagement is still being underestimated as a way to empower patients and determine the circumstances of their daily lives. Participants stated that this is not only the case within patient-caregiver interpersonal relationships, but also within health care organizations and even in the health care system in general. According to participants, one of the ways to improve empowerment is to involve patients in the process of taking responsibility for their own wellbeing and for health care professionals to discuss the right information for shared decision making within a trusted relationship. In addition, participants also mentioned the need for patients and communities to become more knowledgeable consumers of health care services, but also more knowledgeable about healthy eating, physical activity, and so on. Furthermore, a participant proposed that the influence of patients and communities need to be strengthened at a strategic level for instance within PHM initiatives.

### Case study: barriers and recommendations for PHM approach to secondary prevention of ASCVD in Belgium

Table 3 summarizes how focus group participants would identify target patients, assess and evaluate the quintuple aims of the PHM approach, stratify risks, select and design the PHM intervention related to secondary prevention of ASCVD in Belgium, and embed this approach in a process of quality improvement. This table shows that, although some PHM building blocks for secondary ASCVD prevention are already in place, progress still needs to be made with respect to developing a supporting data and knowledge infrastructure; collecting and exchanging data between health care providers; selecting appropriate outcome indicators; establishing collaborative relationships between health care providers; and establishing a ‘learning health system’ culture.

**Table 3:** Population health management approach to tackle secondary prevention in patients with ASCVD in Belgium

	*Intervention*	*Barriers*	*Recommendations*
Population identification	Identify patients with clinically relevant atherosclerosis or atherothrombosis in coronary, carotid or peripheral vascular beds	Limitations of current ICT infrastructure with respect to communication between electronic medical records, data quality and linkage Poor cooperation between software platforms used in different hospital networks and between software platforms used in first and second line Exchange of structured data between first and second line is not automated and requires manual input of data In current fee-for-service system, health care professionals could be perceived to have conflict of interest when identifying patients and inciting them to make appointment	Identify patients in three ways: (a) referral from hospital or from GP practice, (b) active tracking in electronic medical record, (c) direct entry Identify patients in GP practice as 90% of patients in Flanders have a global medical dossier with a GP Identify patients by linking community pharmacy files with electronic medical record Change to bundled payment system in which health care professionals are responsible for following up patients’ health
Quintuple aim assessment	Gain insight into health (inequalities) and wellbeing of target population, costs and quality of care	Diverse databases exist, but are neither linked nor easily accessible Data become available following substantial time delay Not all relevant data are routinely collected	Set up data warehouse based on ICHOM standard sets of outcome indicators for coronary artery disease and stroke [43] or other international indicators and national indicators that can be extracted from electronic medical record Draw on PaRIS project [44] which collects data on patient-reported outcome and experience measures
Risk stratification and intervention selection	Use secondary prevention cardiovascular risk calculators to further subdivide target population according to ASCVD risk Use Kaiser Permanente pyramid to guide interventions	Some data required to enter into risk calculators is missing There are no pre-defined routine cardiovascular risk management follow-up appointments in Belgium	Use data from “Gezondheidsgids” [45] to give individual advice and build action plan with patient Register individual patient goals in electronic medical record Encourage patient co-responsibility and empowerment
Citizens-centered interventions	Design interventions according to 2021 ESC prevention guidelines [46]	Lack of implementation of guidelines in real-life clinical practice	Establish collaborative network between specialist and GP, involving standard communication, peer consultation and referral agreements Decide on intervention based on secondary prevention cardiovascular risk calculators Move to learning health system by translating guidelines into real-life quality indicators Use data about patient lifestyle from “Gezondheidsgids” [45] Train health care professionals in how to attain behavioural change in patients Guide interventions (stimulate self-management, disease management or case management) based on Kaiser Permanente stratification of the population
Quintuple aim impact evaluation	Evaluate impact of intervention with respect to quintuple aims and benchmark with peers	Diverse databases (with data at aggregated or individual patient level) exist, but are not linked Data become available following substantial time delay Not all relevant data are routinely collected	Set up system which automatically extracts relevant data from electronic medical record Bring different data suppliers together to discuss the data needed in a population dashboard to assess and evaluate quintuple aim indicators Develop indicators and benchmarks based on consensus between GPs, cardiologists, nurses, health insurance fund and patient representatives
Quality improvement process	Implement continuous learning cycles focusing on all steps elaborated above	Lack of ‘learning health system’ culture	Prepare checklist of questions, including for example: - Can you make clear which patients are included in the care pathway? - Have you agreed a protocol for diagnosis, working agreements and division of tasks? - Do you have patient referral agreements in place with other primary/secondary care providers? - Does your practice meet ASCVD indicators? Are there any areas for improvement?

Notes: ASCVD = atherosclerotic cardiovascular disease; ESC = European Society of Cardiology; GP = general practitioner; ICHOM = International Consortium for Health Outcomes Measurement; ICT = information and communications technology

## Discussion

This study has explored the current main barriers and recommendations for PHM development in Belgium as voiced by high-level decision makers in medicine, policy and science. The identification of these barriers and recommendations should act as a call-to-action for federal authorities, federated entities and local authorities as well as for providers and other stakeholders (including patients/citizens) to work towards a learning health system that delivers health and wellbeing. Building on the barriers and recommendations formulated by the sampled high-level decision makers, a roadmap is presented below involving multiple steps to implement PHM in Belgium.

### Encourage a sense of urgency to speed up PHM development

It is expected that health care spending will double in Belgium by 2040 and even if finances would allow for it, the manpower to provide the right care and support is lacking [20]. The recent crisis regarding the COVID-19 pandemic has strained the health care system even further [27]. In response to this, the federal authorities, federated entities and local authorities have not been sufficiently able to build on a sense of urgency as a driver for change towards a sustainable health and wellbeing system. This is why a call-to-action in Belgium alike these in other countries such as the Netherlands [5] and England [15] is needed at all levels to speed up PHM development.

### Develop a learning health system embedded in a learning environment supported by a data and knowledge infrastructure

The different levels of government together with all stakeholders need to enforce a learning health system embedded in an evidence-based learning environment that connects the national, regional and individual levels in order to stimulate PHM development by bringing the actual needs to the surface and tackling systemic problems [5, 28–30]. In Flanders, the recently created primary care zones (similar to what is generally known as ‘district health systems’ [31]) may be particularly well suited to advance PHM. Primary care zones plan and coordinate health care and welfare for around 100,000 inhabitants and are expectedto make an assessment of their region by the summer of 2022 in order to identify the ‘needs’ of the population, on the basis of which a policy plan should be drawn up for the following years [32–34]. Tools such as visual dashboards can support this exercise [12].

However, financial investments, knowledge and time are necessary to speed up the development of a learning environment in addition to a data and knowledge infrastructure that contributes to a population-oriented improvement cycle and accountability across organisations and sectors [5]. Such ICT infrastructure and knowledge management can draw on data sources that are already in place in the Belgian health care system such as electronic health records and mandatory electronic prescriptions of medicines [26]. In addition to quantitative data, the data and knowledge infrastructure should also be able to capture qualitative data about for example contextual, social and cultural issues based on a deep understanding of the local community.

Recently, an initiative to bring data suppliers and stakeholders together was launched in Belgium with a view to setting up an accessible and regional data and knowledge infrastructure. This mirrors practices in other countries. In the United States, the Centres of Medicare and Medicaid together with a network of scientific institutions and practice leaders support PHM initiatives with regard to data and technological problems amongst other things [35]. In PHM initiatives, such as Generation Health and Gesundes Kinzigtal, investments in a learning environment including a data and knowledge infrastructure were done by private companies which served as conveners for the initiative [5, 30].

Furthermore, the question can be raised who should take the lead in the further development of a learning environment. PHM initiatives could make agreements on issues such as privacy, indicators, and interdependence and governance of the data and knowledge infrastructure. These initiatives could also make agreements with regard to which stakeholder is going to control this infrastructure as well as about possible investments from the private sectors. It is our opinion that some of these issues, such as the right indicators and data-sharing in relation to the privacy law, need to be addressed at the national level with the involvement of the Belgian government, the federal public service of Health, Food Chain Safety and Environment, and knowledge institutions.

### Reduce uncertainties to enable investments in new payment models and set up new pilots

There are still many questions about new forms of payment, such as bundled payment or a population-based payment [5, 36]. With regard to a population-based payment for instance, a future scenario might be that new legal entities, also described as integrators, are willing to take full responsibility for the financial risks of total health care costs of a regional population [36]. However, there is no decisive answer yet as to whether a population-based payment model actually will provide the social value it is intended to deliver [5]. An alternative that does not require adjustments of the current way of payment is adding value-driven incentives that benefit the health of the population, the so-called mixed payment model [37]. Until now, little is known regarding which novel form of payment is best for which situation, and numerous strategies can be used. Therefore, we recommend that the national government reduces uncertainties such as information asymmetry that hinder the development of new payment models, and sets up new payment model pilots in addition to encouraging knowledge development and sharing of information about these new payment models.

### Start from a joint population-oriented vision

If a sense of urgency for PHM development is lacking in practice, a step-by-step approach based on what is already achieved in a region is important [5, 38]. In addition, further investments in preconditions are necessary such as the right regional leadership of the PHM initiative which will stimulate joint support of regional stakeholders for the initiative based on a shared population-oriented vision [5, 38]. For PHM initiatives whose initiators are primarily from the care sector, it is important not to start an initiative with a broad regional plan or based on payment reforms without such preconditions, as initiators might risk loosing their investments without gaining enough paybacks [5, 38]. As a result, support for the PHM initiative could decrease.

### Enforce collaborative relationships and joint responsibility, including the regional population

Collaborative ownership and responsibility are needed from all stakeholder groups, as progress in PHM cannot be achieved by any one sector or organisation alone [4, 5, 35, 39, 40]. In addition to stakeholders within health and social care, a multi-sectoral perspective is required with input from patients/citizens [41] and wider public services such as education and businesses, financiers, knowledge institutions and regional and national governments. This serves to build up trust and to enter into new collaborative networks that are based on developing health and wellbeing for the population and a learning health system [4, 5]. An example of such a network approach in Belgium is the integrated care (including social care) initiatives which manage chronic patients in a geographical area [21, 42].

### Establish and take an active part in a regional community of practice and put complex problems on the agenda at the federal and regional level

The development of PHM is complex and therefore takes time to evolve, as it requires investments from a diversity of regional stakeholder organizations across health and social care and community services, as well as involvement of patients/citizens [5, 18, 41]. It is important to be in continuous dialogue within communities of practice, and to hold each other accountable in the interest of the population. In addition, developing and exchanging knowledge between regions as well as between the national and regional governance level is important to address complex problems that go along with PHM development and to put these on the agenda [5].

This study is subject to limitations. Our results originate from only two focus group discussions involving 11 participants. However, we were able to recruit key high-level decision makers who have experience with PHM or who are likely to be involved in shaping PHM in Belgium in the future. Also, we feel that the discussions addressed the major guiding principles, building blocks, and key activities that shed light on the underlying barriers and recommendations for PHM in the Belgian context.

## Conclusions

It is clear that Belgium still has a long way to go towards PHM. Interaction between medicine, policy and science, and interaction between federal and regional levels are pivotal for the stimulation of PHM. Next steps should focus on developing a joint population-oriented vision, cultivating shared responsibility for the health of the population, encouraging collaborative relationships (at and between all levels) to gather and analyse data, building a learning health system supported by an environment and infrastructure that facilitate knowledge development and data sharing, experimenting with new policies and payment models, and installing regional PHM initiatives with a focus on patient and population needs.

## Data Availability

The datasets analyzed during the current study are available from the corresponding author on reasonable request.
